# 11β-Hydroxysteroid dehydrogenase type 2 may mediate the stress-specific effects of cortisol on brain cell proliferation in adult zebrafish (*Danio rerio*)

**DOI:** 10.1242/jeb.248020

**Published:** 2024-08-27

**Authors:** E. Emma Flatt, Sarah L. Alderman

**Affiliations:** Department of Integrative Biology, University of Guelph, 50 Stone Road East, Guelph, ON, Canada, N1G 2W1

**Keywords:** Stress biology, Neuroendocrinology, Neuroplasticity, Regulation of mitosis, Adult neurogenesis, Glucocorticoid regulation

## Abstract

Stress-induced increases in cortisol can stimulate or inhibit brain cell proliferation, but the mechanisms behind these opposing effects are unknown. We tested the hypothesis that 11β-hydroxysteroid dehydrogenase type 2 (Hsd11b2), a glucocorticoid-inactivating enzyme expressed in neurogenic regions of the adult zebrafish brain, mitigates cortisol-induced changes to brain cell proliferation, using one of three stress regimes: a single 1 min air exposure (acute stress), two air exposures spaced 24 h apart (repeat acute stress) or social subordination (chronic stress). Plasma cortisol was significantly elevated 15 min after air exposure and recovered within 24 h after acute and repeat acute stress, whereas subordinate fish exhibited significant and sustained elevations relative to dominant fish for 24 h. Following acute stress, brain *hsd11b2* transcript abundance was elevated up to 6 h after a single air exposure but was unchanged by repeat acute stress or social subordination. A sustained increase in brain Hsd11b2 protein levels occurred after acute stress, but not after repeat or chronic stress. Following acute and repeat acute stress, brain *pcna* transcript abundance (a marker of cell proliferation) exhibited a prolonged elevation, but was unaffected by social subordination. Interestingly, the number of telencephalic BrdU+ cells increased in fish after a single air exposure but was unchanged by repeat acute stress. Following acute and repeat acute stress, fish expressed lower brain glucocorticoid and mineralocorticoid receptor (*gr* and *mr*) transcript abundance while subordinate fish exhibited no changes. Taken together, these results demonstrate stressor-specific regulation of Hsd11b2 in the zebrafish brain that could modulate rates of cortisol catabolism contributing to observed differences in brain cell proliferation.

## Teaser

**Summary:** The steroid dehydrogenase Hsd11b2 is dynamically regulated in the zebrafish brain during stress: variation in Hsd11b2 abundance may underscore stress-specific changes in forebrain neurogenesis.

## INTRODUCTION

Adult neurogenesis is the formation of new functional neurons from neural stem and progenitor cells (NSPCs) in the postnatal brain. Adult neurogenesis in the forebrain has been described in at least one species of each vertebrate class (Kaslin et al., 2008; Zupanc, 2021); however, considerable variation in the number of neurogenic niches and the rate of proliferation exists across taxa. Teleost fish have emerged as important model species in this field because of their comparatively greater capacity for lifelong neurogenesis (Zupanc, 2021). In the zebrafish (*Danio rerio*), for example, neurogenic niches are not restricted to the forebrain, but occur along the entire rostro-caudal axis of the brain (Zupanc et al., 2005) with a relatively high rate of NSPC proliferation (Hinsch and Zupanc, 2007) and niche-specific regulation of neurogenic activity (Caron et al., 2022). The functional significance of adult neurogenesis in teleost fish is an area of active study, but it is widely accepted to have both additive and reparative roles, i.e. supporting indeterminant growth (Zupanc and Horschke, 1995; Traniello et al., 2014) and injury repair (März et al., 2011; Lefevre et al., 2017), respectively. In addition, evidence that lifelong neurogenesis in the telencephalon contributes to learning and memory in fish (Ausas et al., 2019; Mazzitelli-Fuentes et al., 2022), as it does in mammals (van Praag et al., 1999; Lemaire et al., 2000; Hairston et al., 2005; Monteiro et al., 2014), songbirds (Hall et al., 2014; Guitar and Sherry, 2018) and reptiles (LaDage et al., 2013), suggests that certain attributes of adult neurogenesis are conserved across vertebrate taxa.

Numerous endogenous factors can alter adult neurogenesis, including the stress-induced release of glucocorticoid hormones. Glucocorticoids are pleiotropic steroid hormones that bind to both glucocorticoid and mineralocorticoid receptors (Gr and Mr) (Wendelaar Bonga, 1997; Mommsen et al., 1999; Aluru and Vijayan, 2009). Both Gr and Mr are widely expressed in the vertebrate brain (Wendelaar Bonga, 2011; Godoy et al., 2018), including in regions where NSPC proliferation occurs. Many studies in mammals suggest that acute transient increases in glucocorticoids stimulate brain cell proliferation (Thomas et al., 2006; Dagyte et al., 2009; Kirby et al., 2013; So et al., 2017), while chronically elevated glucocorticoids inhibit brain cell proliferation (Gould et al., 1998; Daygte et al., 2009). Research in fish supports a similar association between plasma cortisol, the principal glucocorticoid in fish, and brain cell proliferation. For example, the expression of *proliferating cell nuclear antigen* (*pcna*; a cell proliferation marker) was upregulated following brief air exposure in the whole brain of black rockfish (*Sebastes schlegelii*; Zhang et al., 2020) and after short-term confinement in the telencephalon of rainbow trout (*Oncorhynchus mykiss*; Johansen et al., 2012), concomitant with an increase in plasma cortisol. Conversely, the sustained increase in plasma cortisol resulting from a cortisol-laced diet or from social subordination reduced cell proliferation in the telencephalon (Sørensen et al., 2011, 2012; Tea et al., 2019). Still, findings that oppose these trends are not uncommon. For example, the transient increase in plasma cortisol following a series of acute stressors (10 min confinement, 1 min air exposure and 5 min chase) was associated with fewer 5′-bromo-2′-deoxyuridine-labelled cells (BrdU+, a thymidine analogue) in the telencephalon of juvenile European sea bass (*Dicentrarchus labrax*) relative to unstressed controls (Fokos et al., 2017), whereas chronically increased plasma cortisol in brown ghost knifefish (*Apteronotus leptorhynchus*) coincided with greater numbers of BrdU+ cells in the periventricular zones compared with fish held in isolation (Dunlap et al., 2006). A further challenge is that not all neurogenic niches respond in the same way to stress. For example, 2 weeks of social isolation reduced whole-body cortisol levels in zebrafish, alongside fewer BrdU+ cells in the periventricular zone of the caudal optic tectum and the vagal lobe of the hindbrain but more BrdU+ cells in the dorsal telencephalon (Lindsey and Tropepe, 2014). Importantly, pharmacological agents that inhibit cortisol synthesis or block Gr have successfully reversed the effects of stress on brain cell proliferation in fish (Dunlap et al., 2011; Tea et al., 2019). Altogether, the results of these studies support a role for cortisol in regulating neurogenesis along a continuum from stimulatory to inhibitory; however, the mechanisms that underpin this variable regulation are unclear.

Intracellular glucocorticoids can be catabolized by the enzyme 11β-hydroxysteroid dehydrogenase type 2 (Hsd11b2), which converts cortisol (humans, fish) or corticosterone (rodents, birds) to their inert forms, cortisone or 11-dehydrocorticosterone, respectively (Chapman et al., 2013). Cortisone is then converted to 20β-hydroxycortisone by 20β-hydroxysteroid dehydrogenase type 2 (Hsd20b2) for excretion (Tokarz et al., 2012, 2013). In addition, the enzyme Hsd11b2 also demonstrates other functions in steroid hormone pathways, including generating the principal fish androgen, 11-ketotestosterone (Tsachaki et al., 2017). At the cellular level, the activity of Hsd11b2 may account for variable responses to cortisol signalling by buffering intracellular cortisol levels and the downstream activation of Gr and Mr. Indeed, previous work in mammals has shown that HSD11B2 in the placenta and in the fetal brain is critical for protecting brain development during maternal stress by preventing glucocorticoid-induced inhibition of neurogenesis (Diaz et al., 1998; Wyrwoll et al., 2015). Intriguingly, Hsd11b2 activity in the brain increased following acute stress in adult zebrafish, and *in situ* hybridization localized *hsd11b2* throughout the brain, including along the perimeter of the olfactory bulbs and dorsal telencephalon as well as lining the telencephalic ventricle (Alderman and Vijayan, 2012). Although co-labelling studies have not been performed to localize Hsd11b2 to specific cell types, the expression of Hsd11b2 in these forebrain regions is consistent with known neurogenic niches (Zupanc, 2006) and supports a role for this enzyme in the neurogenic process. Therefore, this study tested the hypothesis that variation in brain Hsd11b2 facilitates the stressor-specific effects of cortisol on brain cell proliferation in zebrafish.

## MATERIALS AND METHODS

### Animals

Adult wild-type zebrafish, *Danio rerio* (Hamilton 1822), were obtained from a local supplier and held at the Hagen Aqualab (University of Guelph, ON, Canada). Fish were maintained at 27°C with a 13 h:11 h light:dark photoperiod and fed twice daily with GEMMA Micro 300 (ZEBCARE B.V., Nederweert, The Netherlands). The use and care of these animals were approved by the University of Guelph's Animal Care Committee in accordance with guidelines of the Canadian Council for Animal Care.

### Experiment 1: stress-specific regulation of *hsd11b2* in the brain

Fish were exposed to one of three stress regimes: a single 1 min air exposure (acute stress), two 1 min air exposures spaced 24 h apart (repeat acute stress) or social subordination (chronic stress). For acute stress and repeat acute stress, a total of 91 adult mixed sex zebrafish were randomly distributed across 14, 2 l tanks (*N*=6–7 per tank; 2 tanks per time point). Following a 1 week acclimation, fish were either immediately euthanized as described below (time 0) or subjected to a standardized 1 min air exposure by simultaneously transferring all fish in a tank to a fish net and holding them in the air for exactly 1 min, as previously described (Alderman and Vijayan, 2012). The fish were then returned to the tank to recover for 15 min, 6 h, 24 h or 48 h. This timing was chosen to encompass the temporal dynamics of cortisol and Hsd11b2 responses to stress (i.e. the rapid rise and recovery of cortisol, and the slower and extended increase in Hsd11b2 activity; Alderman and Vijayan, 2012). The fish from four experimental tanks underwent a second 1 min air exposure at 24 h followed by recovery for 15 min or 24 h (repeat acute stress) to determine whether any changes in Hsd11b2 expression would be augmented or suppressed by a second stressor. At the determined recovery time, all fish in a tank were rapidly and simultaneously killed using an overdose of ice-cold buffered tricaine methane sulfonate (MS-222; 0.3 g l^−1^ MS-222, 0.6 g l^−1^ NaHCO_3_). The tail was severed, and blood collected by gravity flow as described by Babaei et al. (2013), followed by blood centrifugation to isolate plasma (*N*=13 per time point). The brains were then removed, snap frozen on dry ice, and stored at −80°C for future analysis of gene (*N*=8 per time point) or protein expression (*N*=5 per time point). Note that for acute and repeat acute stress gene expression only, the brains were bisected into two zones at the boundary of the optic tectum to examine region-specific changes. Brain zone 1 included the telencephalon and olfactory bulbs, and brain zone 2 included all remaining brain regions (optic tectum, diencephalon, hypothalamus, cerebellum, rhombencephalon, medulla). Brain zone 1 was isolated as a region of interest as it displays high levels of neurogenic activity (Zupanc, 2006) and Hsd11b2 expression (Alderman and Vijayan, 2012). However, low total RNA yield required pooling brain zone 1 samples (*N*=4 samples, where each sample is from 2 individual fish); therefore, all subsequent experiments and endpoints used whole-brain homogenates.

For chronic stress, a total of 24 adult male zebrafish were anaesthetized using MS-222 (0.15 g l^−1^ MS-222, 0.3 g l^−1^ NaHCO_3_) and fin-clipped for identification. Fish were size matched based on mass (mean dominant 0.80±0.02 g, mean subordinate 0.81±0.02 g, mean difference between pairs 0.006±0.003 g) and fork length (mean dominant 4.22±0.04 cm, mean subordinate 4.27±0.04 cm, mean difference between pairs 0.04±0.20 cm). Each pair of fish was acclimated for 24 h in a 2.6 l tank, with one individual on either side of a perforated opaque divider. Each experimental tank contained an air stone and a constant inflow of filtered well water at ∼27°C supplied on both sides of the divider. The barrier was then removed, and the fish were allowed to interact for 5 min to establish the dominant–subordinate hierarchy. The hierarchy was considered to have formed when one fish started to perform retreats and ceased using aggressive behaviours. The behaviour of each fish was then recorded using an established scoring system (Filby et al., 2010; Paull et al., 2010; Jeffrey and Gilmour, 2016; Tea et al., 2019) that considered acts of aggression (biting, chasing), number of retreats, position in the tank, and order of feeding during a 5 min observation period. The fish were killed after 24 h of interaction, and tissues collected as above. This timing was chosen based on Tea et al. (2019), where reduced forebrain cell proliferation was observed in subordinate fish injected with BrdU after 24 h of social interaction, and to permit comparison with the 24 h recovery time point of the acute and repeat acute stress experiment. A separate cohort of fish was directly sampled from group housing to serve as a control group (*N*=13).

### Experiment 2: effect of acute and repeat acute stress on cell proliferation in the telencephalon

A total of 12 adult mixed sex zebrafish were randomly distributed across three 2 l tanks (*N*=4 per tank) and acclimated for 1 week. Following acclimation, fish in an experimental tank were either netted and immediately anaesthetized (time 0) or subjected to a single or repeated 1 min air exposure (see experiment 1) before undergoing light anaesthesia by gradual cooling to 12°C. Each fish was given a single intraperitoneal injection of BrdU (Sigma-Aldrich, St Louis, MO, USA; 40 µl g^−1^ body mass) and then allowed to recover for 1.5 h (timing informed by results of experiment 1). The fish were killed as above and the brains prepared for cryosectioning exactly as previously described (Tea et al., 2019). Brains were cryosectioned at 20 µm and every third section throughout the telencephalon was kept for immunohistochemistry [see below; 12 sections per fish, collected on Fisherbrand Superfrost Plus slides (Thermo Fisher Scientific, Waltham, MA, USA) and frozen at −20°C], encompassing approximately levels 50–92 (Wullimann et al., 1996) based on gross anatomical features.

### Plasma cortisol

Plasma was thawed on ice, diluted 30 times with 1× assay buffer and used in duplicate reactions to quantify cortisol concentration using a commercially available enzyme-linked immunosorbent assay (ELISA; Neogen, Lexington, KY, USA) exactly according to the manufacturer’s instructions. Samples that fell outside the range of the standard curve could not be re-assayed because of limited plasma availability and were therefore removed from further analysis. Intra-assay variation was 3.3% across all plates. Inter-assay variation was 9.5% for acute stress samples; all chronic stress samples were assayed on a single plate.

### Western blot: quantifying Hsd11b2 protein abundance

Total protein was extracted from whole-brain samples by homogenizing in 100 µl of 1× RIPA buffer containing protease inhibitors (cOmplete mini protease inhibitor cocktail, Hoffmann La Roche Limited, Mississauga, ON, Canada) and prepared for western blotting exactly as previously described (Dindia et al., 2017). For each sample, 20 µg total protein was loaded on a 10% SDS-polyacrylamide gel, separated by SDS-PAGE and transferred to 0.45 µm nitrocellulose membrane (Bio-Rad Laboratories Ltd, Montreal, QC, Canada; 30 V×16 h). Each gel contained a loading control (pooled brain homogenate) and all representative time points (acute, repeat) or social statuses (chronic) for a given experiment. Following transfer, the membranes were blocked in 1× fish gelatin blocking buffer (Biotium Inc., Fremont, CA, USA) for 1 h at room temperature and then incubated overnight at 4°C in chicken anti-Hsd11b2 (Genetel, Madison, WI, USA) diluted to 0.5 µg ml^−1^ in 2% skimmed milk prepared in Tris-buffered saline containing 0.05% Triton X-100 (TBST). The primary antibody was custom designed to recognize a conserved region of rainbow trout and zebrafish Hsd11b2 (Best et al., 2023), and was a generous gift from Dr Kathleen M. Gilmour (University of Ottawa, Canada). The antibody was fully validated for specificity and linear quantification prior to use in these experiments (Fig. S1). Immunodetection was completed with a 1 h incubation in HRP-conjugated secondary antibody [goat anti-chicken IgY H&L (HRP); cat. no. ab97135, Abcam, Cambridge, UK] diluted to 0.13 µg ml^−1^ in 2% skimmed milk in TBST, followed by chemiluminescent detection using Clarity Western Enhanced Chemiluminescence Substrate (Bio-Rad) on a ChemiDoc MP Imaging System (Universal Hood III, Bio-Rad). The membranes were then stripped (2 mmol l^−1^ glycine, 0.1% sodium dodecyl sulphate, 1% Tween 20; pH 2.2) for 10 min at room temperature and washed (2×10 min in phosphate-buffered saline, PBS), 1×5 min in TBST, 1×5 min in TBS) prior to total protein staining using SYPRO Ruby Protein Blot Stain (Invitrogen Canada Inc., Burlington, ON, Canada) according to the manufacturer's instructions. The protein abundance of each sample was quantified as band intensity (arbitrary units) using Image Lab Software version 6.1 (Bio-Rad) and normalized to total protein.

### Quantitative reverse transcription-PCR (RT-qPCR)

Total RNA was extracted from frozen brain tissue using TRIzol Reagent (Invitrogen) following the manufacturer’s instructions. The purity and concentration were verified (NanoDrop 2000 Spectrophotometer; Thermo Fisher Scientific, Waltham, MA, USA) and then 1 µg of total RNA was treated with DNase I prior to cDNA synthesis using the High-Capacity cDNA Reverse Transcription kit (Applied Biosystems, Foster City, CA, USA), all according to the manufacturers' instructions. To test the efficacy of the DNase treatment, non-reverse transcribed (non-RT) controls were included for a subset of random samples (∼10%) by omitting the MultiScribe RT enzyme in the cDNA synthesis reaction. qPCR was used to quantify the gene expression of *elongation factor 1α* (*ef1α*), *ribosomal protein L8* (*rpl8*), *hsd11b2*, *pcna*, *gr* and *mr* separately in duplicate 15 µl reactions containing 1× Sso Advanced Universal SYBR Green Supermix (Bio-Rad), the gene-specific primer pair (Table 1) and 5 µl of template (diluted cDNA, non-RT control or water). Cycling conditions were as specified by the manufacturer and included a final melt curve. Mean cycle threshold (Ct) values were fitted to the antilog of standard curves generated for each primer pair using serially diluted cDNA. The transcript abundance of each gene of interest was then normalized to the mean expression of two housekeeping genes, *ef1α* and *rpl8*, which were stably expressed across all treatments (one-way analysis of variance, ANOVA).

**Table:**
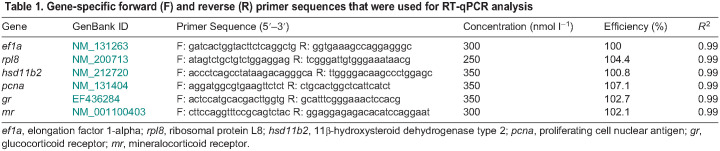
Table 1. Gene-specific forward (F) and reverse (R) primer sequences that were used for RT-qPCR analysis

### Immunohistochemistry

Slides were thawed at room temperature and then rehydrated in PBS prior to antigen retrieval in 10 mmol l^−1^ sodium citrate buffer (pH 6) with 0.05% Tween-20 at 65°C for 30 min. Slides were cooled to room temperature for 15 min, then immersed in 2 mol l^−1^ HCl for 15 min followed by 2×5 min washes in 0.1 mol l^−1^ borate buffer (pH 8.5). The sections were permeabilized for 3×5 min in PBS containing 0.05% Triton X-100 (PBST), and then blocked with 5% goat serum (Invitrogen) in PBST for 45 min at room temperature. BrdU-labelled cells were visualized using mouse anti-BrdU [cat. no. G3G4 (anti-BrdU), Developmental Studies Hybridoma Bank, Iowa City, IA, USA] diluted to 1:100 in blocking buffer (overnight, 4°C) and Alexa Fluor 488-conjugated goat anti-mouse IgG (cat. no. 115-545-003, Jackson ImmunoResearch, West Grove, PA, USA) for 3 h at room temperature in the dark. Sections were imaged using a Nikon ECLIPSE Ti2 (Nikon Instruments Inc., Melville, NY, USA). All BrdU+ cells on the surface (dorsal, midline and ventral) of each section were tallied and normalized to cross-sectional area (µm^2^), which was stable across all experimental groups (one-way ANOVA).

### Statistical analysis

Social rank was assigned based on the behavioural scores of each fish using a principal components analysis (PCA): low behaviour scores were associated with dominance (PC1 eigenvalue=1.7). Therefore, within each pair, the fish with the lower behaviour score was assigned dominant status (mean±s.e.m. behaviour score=−1.65±0.05) and the fish with the higher score was assigned subordinate status (mean±s.e.m. behaviour score=1.65±0.06).

Differences in plasma cortisol, normalized mRNA abundance (*hsd11b2*, *pcna*, *gr, mr*) and Hsd11b2 protein expression between recovery time points (acute stress, repeat acute stress) or between social status categories (chronic stress), as well as brain cell proliferation (BrdU+ cells µm^−2^) were determined using one-way ANOVA followed by Tukey's *post hoc* tests. Data that did not meet the assumptions of normality (Shapiro–Wilk test) or equal variance (Bartlett test) were either log transformed or analysed using non-parametric tests (Kruskal–Wallis followed by Dunn's *post hoc* test). Because of low RNA yield in brain zone 1 samples, *gr* and *mr* mRNA abundance could not be quantified at 6 h recovery from acute stress. Any data points greater than or less than 1.5× the interquartile range from the upper or lower quartile were removed prior to statistical analysis (no more than two outliers were removed from any treatment group). For transparency, all outliers are indicated on graphs as black triangles. All statistical analyses were performed in R Statistical Software (v4.2.2; http://www.R-project.org/) and α was set to 0.05. Figures were produced using GraphPad Prism version 10.2.0 (GraphPad Software, Boston, MA, USA).

## RESULTS

### The effects of stressor exposure on plasma cortisol

**Fig. 1.**
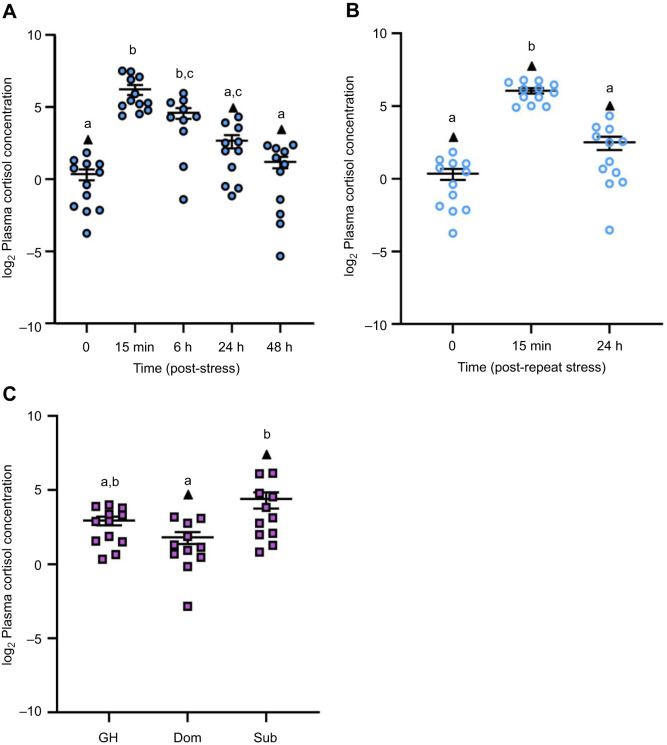
**The effects of stress exposure on plasma cortisol.** (A) Zebrafish were exposed to air for 1 min (stressor) and allowed to recover for up to 48 h (filled blue circles). (B) For a separate group of fish, the stressor was repeated 24 h after the first stressor and fish were allowed to recover for 15 min or 24 h (open blue circles). (C) Differences in the plasma cortisol of dominant (Dom) and subordinate (Sub) zebrafish after 24 h of social interaction; group-housed (GH) fish are included to act as a control (filled purple squares). Data points identified as statistical outliers were removed prior to analysis and are shown as black triangles. Each symbol represents the cortisol concentration (ng ml^−1^) of an individual fish, with a solid line and whiskers representing the mean±s.e.m. (acute stress *n*=10–13, repeat acute stress *n*=12, social stress *n*=11–13). Differences in plasma cortisol levels were determined by one-way ANOVA and Tukey's *post hoc* test, except for repeat acute stress, where a Kruskal–Wallis and Dunn's *post hoc* test was used (*P*<0.05). Within each panel, groups that do not share a common letter are significantly different from one another.

Plasma cortisol levels changed over time after zebrafish were exposed to a 1 min air exposure stressor (*F*_4,51_=20.17, *P<*0.0001; Fig. 1A). At 15 min post-stress, fish experienced a 75-fold increase in plasma cortisol levels relative to baseline (*P*<0.0001). At 6 h post-stress, plasma cortisol levels remained elevated relative to baseline (*P<*0.0001) but returned to pre-stress levels by 24 h (*P*>0.5). Similarly, repeat acute stress exposure induced a 48-fold increase in plasma cortisol levels at 15 min recovery relative to baseline (*H*=25.297, *P*<0.0001, d.f.=2; Fig. 1B), with complete recovery to baseline by 24 h (*P=*0.0002).

Plasma cortisol levels were affected by social status (*F*_2,31_=5.528, *P*=0.0090; Fig. 1C). Subordinate fish experienced a 6.3-fold increase in plasma cortisol levels relative to dominant fish (*P*=0.0073). Plasma cortisol of group-housed fish was intermediate between that of dominant and subordinate fish (*P*>0.05).

### The effects of stress and recovery on *hsd11b2* expression in the brain

**Fig. 2.**
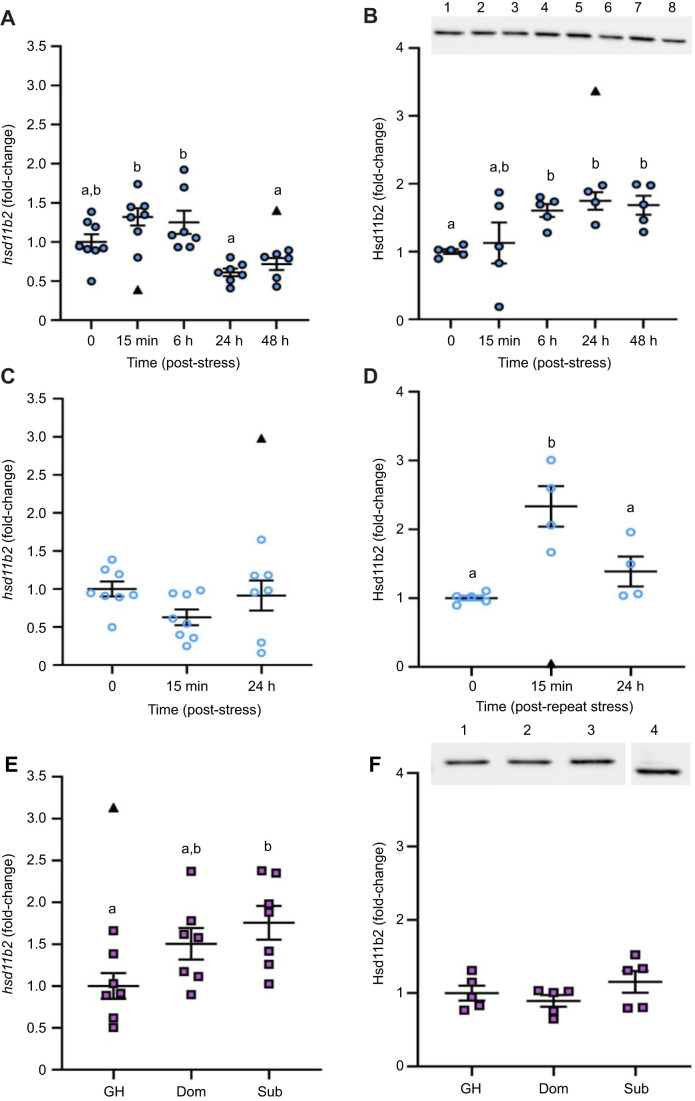
**The effects of stress on *hsd11b2* mRNA and Hsd11b2 protein abundance in the adult zebrafish brain.** Changes in brain *hsd11b2* transcript and Hsd11b2 protein abundance following an acute stressor (A,B), a repeat acute stressor (C,D) and a chronic stressor (E,F). Gene expression was quantified either in brain zone 2 (telencephalon and olfactory bulbs removed; A,C) or in whole brain (E). In B, a representative western blot for acute and repeat acute stress is provided (lane 1=0 min, 2=15 min, 3=6 h, 4=24 h and 6=48 h post-acute stress; lane 5=15 min, 7=24 h post-repeat acute stress; lane 8=internal control). The full blots are provided in Fig. S2. In F, a representative western blot for chronic stress is provided [lane 1=group housed (GH), 2=dominant (Dom), 3=subordinate (Sub), 4=internal control from the same blot]. Transcript abundance was normalized to the mean expression of the two housekeeping genes (*ef1α* and *rpl8*). Protein expression of Hsd11b2 was normalized to total protein. Data are shown as individual data points with a solid line and whiskers representing the mean±s.e.m. (*hsd11b2*, acute stress *n*=6–8, repeat acute stress *n*=7–8, social stress *n*=7; Hsd11b2, acute stress *n*=4–5, repeat acute stress *n*=4–5, social stress *n*=5). Data points identified as statistical outliers were removed prior to analysis and are shown as black triangles. Differences in *hsd11b2* mRNA and Hsd11b2 protein levels were determined by one-way ANOVA followed by a Tukey's *post hoc* test, except for Hsd11b2 protein abundance under acute stress (B), where a Kruskal–Wallis followed by a Dunn's *post hoc* test was used (*P<*0.05). Within each panel, groups that do not share a common letter are significantly different from one another.

Temporal changes in *hsd11b2* mRNA abundance in brain zone 2 (*F*_4,30_=8.952, *P*<0.0001; Fig. 2A) and Hsd11b2 protein expression in whole brain (*H*=11.403, *P*=0.0224, d.f.=4; Fig. 2B) were observed following 1 min air exposure stress. The mRNA abundance of *hsd11b2* was unchanged at 15 min and 6 h recovery (*P*>0.05). After 24 h and 48 h recovery, *hsd11b2* mRNA abundance was 2-fold lower than at 15 min (*P=*0.0004 and *P*=0.0036) and 6 h post-stress (*P=*0.0012 and *P*=0.0110). Similar trends in *hsd11b2* mRNA abundance were found in the pooled brain zone 1 samples, although it did not reach statistical significance (*F*_4,12_=2.63, *P*=0.0870; Fig. S3A). Brain Hsd11b2 protein abundance was 1.7-fold higher than pre-stress levels by 6 h recovery (*P*=0.0142) and remained elevated through to 48 h post-stress (*P*=0.0051). In contrast, repeat acute stress exposure did not significantly affect *hsd11b2* mRNA abundance in either brain zone (*F*_2,20_=2.192, *P*=0.1378; *F*_2,7_=2.107, *P=*0.1920, respectively; zone 2, Fig. 2C; zone 1, Fig. S3B); however, whole-brain Hsd11b2 protein abundance was affected (*F*_2,10_=15.12, *P=*0.0009; Fig. 2D). Specifically, Hsd11b2 was 2.3-fold higher than baseline at 15 min recovery (*P*=0.0008) but returned to pre-stress levels by 24 h recovery.

Social status had differential effects on whole-brain *hsd11b2* mRNA abundance (*F*_2,18_=4.48, *P=*0.0264; Fig. 2E). Although *hsd11b2* mRNA abundance was similar between dominant and subordinate fish, it was 1.8-fold higher in subordinate fish relative to group-housed fish (*P=*0.0260). There were no significant differences in whole-brain Hsd11b2 protein abundance between dominant, subordinate or group-housed fish (*F*_2,12_=1.321, *P*=0.3030; Fig. 2F).

### The effects of stress and recovery on brain cell proliferation

**Fig. 3.**
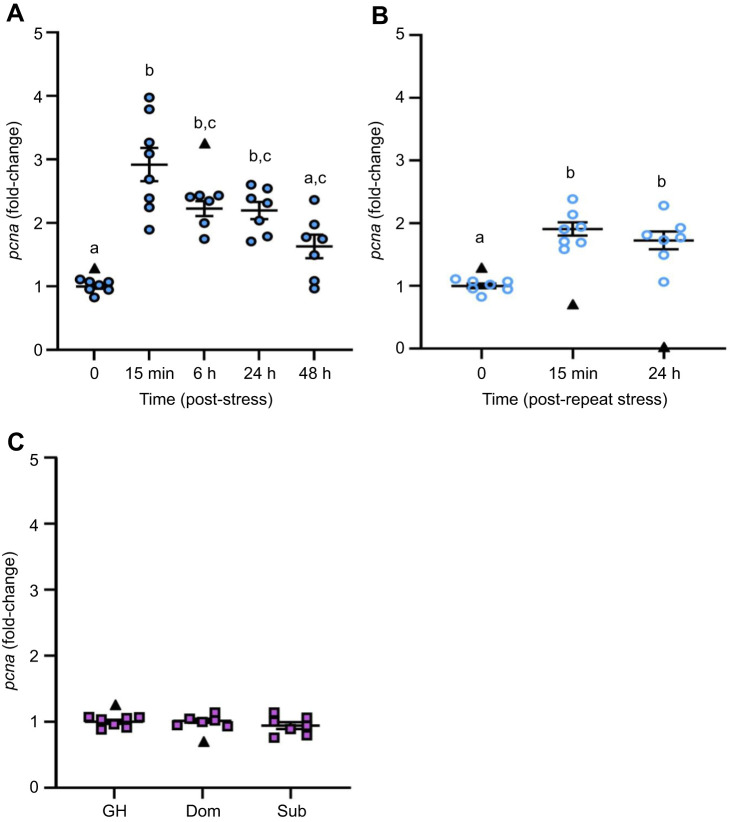
**The effects of stress on *pcna* transcript abundance in the adult zebrafish brain.** (A) Zebrafish were exposed to air for 1 min and allowed to recover for up to 48 h (filled blue circles). (B) For a separate group of fish, the stressor was repeated 24 h after the first stressor and fish were allowed to recover for 15 min or 24 h (open blue circles). (C) Dominant (Dom) and subordinate (Sub) zebrafish after 24 h of social stress; group-housed (GH) fish are included as a control (purple squares). *pcna* transcript abundance in brain zone 2 (telencephalon and olfactory bulbs removed; A,B) and whole brain (C) was normalized to the mean expression of the two housekeeping genes (*ef1α* and *rpl8*). Data are shown as individual data points with a solid line and whiskers representing the mean±s.e.m., and statistical outliers as black triangles. Differences in *pcna* transcript abundance were determined by one-way ANOVA with a Tukey's *post hoc* test, except for acute stress, where a Kruskal–Wallis with a Dunn's *post hoc* test was used (*P*<0.05). Within each panel, groups that do not share a common letter are significantly different from one another.

*pcna* mRNA abundance in brain zone 2 was significantly different among time points following acute stress exposure (*H*=23.346, d.f.=4, *P*=0.0001; Fig. 3A). The abundance of *pcna* was 3-fold higher than pre-stress levels after 15 min recovery (*P*<0.0001) and remained elevated through to 24 h recovery (*P*=0.0199) but returned to baseline by 48 h recovery. Similarly, *pcna* mRNA abundance in brain zone 1 was significantly affected by acute stress exposure (*H*=9.691, *P*=0.0460, d.f.=4; Fig. S4A). Here, *pcna* mRNA abundance showed a 5-fold increase at 15 min post-stress (*P=*0.0019) but returned to baseline levels by 6 h recovery. Repeat acute stress also affected *pcna* mRNA abundance in brain zone 2 (*F*_2,18_=27.78, *P*<0.0001; Fig. 3B), being at least 1.7-fold higher than baseline levels at 15 min and 24 h recovery (*P<*0.0001). A similar trend was found in brain zone 1, where *pcna* mRNA abundance was 2-fold higher at 15 min and 24 h post-repeat acute stress relative to baseline, but this did not reach statistical significance (*H*=5.6, *P*=0.0608, d.f.=2; Fig. S4B). Social status did not affect whole-brain *pcna* mRNA abundance (*F*_2,17_=0.943, *P*=0.4090; Fig. 3C).

**Fig. 4.**
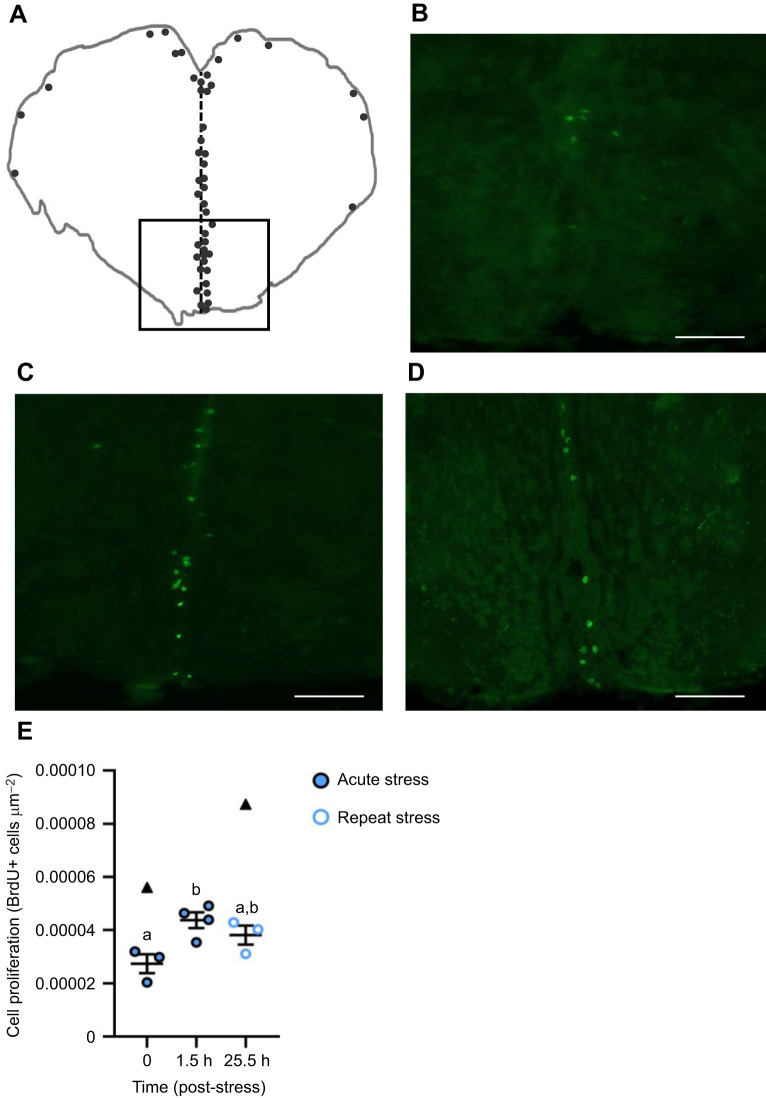
**The effects of a single and repeat acute stressor on brain cell proliferation in the telencephalon of adult zebrafish brain.** (A) A line drawing traced from original images of cross-sections following immunohistochemical detection, where the vertical dashed line represents the midline of the section and circles represent common locations of BrdU+ cells. The approximate corresponding level in the zebrafish brain atlas is the rostral telencephalon level 71 (Wullimann et al., 1996), based on gross anatomical features across the suite of serial sections for each represented fish. The boxed region indicates the area of the representative photomicrographs of cross-sections through the zebrafish telencephalon of (B) time 0 (baseline), (C) 1.5 h recovery following a single 1 min air exposure stressor and (D) 1.5 h recovery from a repeat stressor 24 h after the initial stressor (i.e. 25.5 h from time 0). Scale bars: 100 µm. To estimate cell proliferation in the telencephalon (E), the number of surface BrdU+ cells (green) in each serial 20 µm section was tallied and normalized to cross-sectional area (µm^2^). Each data point is the sum tally from 12 serial sections per fish, with a solid line and whiskers representing the mean±s.e.m. for each time point, and statistical outliers shown as black triangles. Changes in cell proliferation following stress exposure were determined using a one-way ANOVA with a Tukey's *post hoc* test (*P*<0.05). Within each panel, time points that do not share a common letter are significantly different from one another.

The number of BrdU+ cells in the telencephalon changed following an air exposure stressor (*F*_2,7_=6.215, *P*=0.0281; Fig. 4). Specifically, the number of BrdU+ cells in the telencephalon was 1.6-fold greater at 1.5 h recovery from the acute stress relative to unstressed controls (*P*=0.0300). Following 1.5 h recovery from the repeat acute stressor, the number of BrdU+ cells in the telencephalon was intermediate between the acute stress-exposed fish and unstressed controls (*P*=0.1300, *P*=0.2700).

### The effects of stress and recovery on the transcript abundance of *gr* and *mr* in the brain

**Fig. 5.**
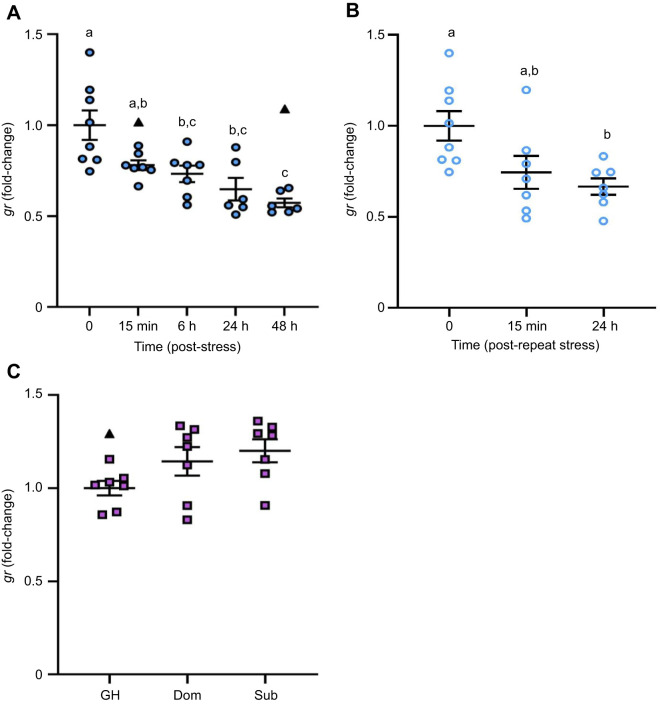
**The effects of stress exposure on *gr* transcript abundance in the adult zebrafish brain.** (A) Zebrafish were exposed to air for 1 min and allowed to recover for up to 48 h (filled blue circles). (B) For a separate group of fish, the stressor was repeated 24 h after the first stressor and fish were allowed to recover for 15 min or 24 h (open blue circles). (C) Dominant (Dom) and subordinate (Sub) zebrafish after 24 h of social stress; group-housed (GH) fish are included as a control (purple squares). *gr* transcript abundance was normalized to the mean expression of two housekeeping genes (*ef1α* and *rpl8*). Data are shown as individual data points with a solid line and whiskers representing the mean±s.e.m., and statistical outliers as black triangles. Changes in *gr* transcript abundance in brain zone 2 (telencephalon and olfactory bulbs removed; A,B) and the whole brain (C) were determined using a one-way ANOVA followed by a Tukey's *post hoc* test (*P<*0.05). Within each panel, groups that do not share a common letter are significantly different from one another.

*gr* transcript abundance was significantly different in brain zone 2 among time points following acute stress exposure (*F*_4,29_=10.02, *P*<0.0001; Fig. 5A). The *gr* mRNA abundance was 1.4-fold lower than pre-stress levels as early as 15 min recovery; however, this decrease did not reach statistical significance until 6 h, 24 h and 48 h (∼1.7-fold, *P*=0.0169, *P=*0.0006 and *P*<0.0001, respectively). The transcript abundance of *gr* in brain zone 1 was significantly affected by acute stress (*H*=9.4762, *P*=0.0236, d.f.=3; Fig. S5A), where a significant decrease in *gr* mRNA abundance was observed at 48 h post-stress relative to baseline (*P*=0.0046). Repeat acute stress significantly affected *gr* mRNA abundance in brain zone 2 (*F*_2,19_=5.524, *P*=0.0128; Fig. 5B), where a significant 1.5-fold decrease in *gr* mRNA abundance occurred at 24 h recovery relative to baseline (*P*=0.0140). In contrast, *gr* mRNA abundance within brain zone 1 was not significantly affected by repeat acute stress (*H*=5.6, *P*=0.0608, d.f.=2; Fig. S5B). Social status did not affect whole-brain *gr* mRNA levels (*F*_2,18_=2.871, *P=*0.0828; Fig. 5C).

**Fig. 6.**
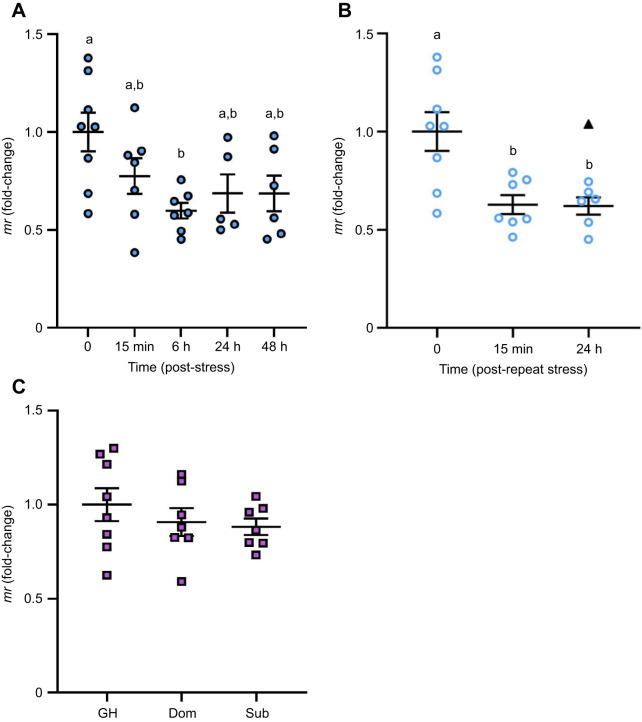
**The effects of stress exposure on *mr* transcript abundance in the adult zebrafish brain.** (A) Zebrafish were exposed to air for 1 min and allowed to recover for up to 48 h (filled blue circles). (B) For a separate group of fish, the stressor was repeated 24 h after the first stressor and fish were allowed to recover for 15 min or 24 h (open blue circles). (C) Dominant (Dom) and subordinate (Sub) zebrafish after 24 h of social stress; group-housed (GH) fish are included as a control (purple squares). *mr* transcript abundance was normalized to the mean expression of two housekeeping genes (*ef1α* and *rpl8*). Data are shown as individual data points with a solid line and whiskers representing the mean±s.e.m., and statistical outliers as black triangles. Changes in *mr* transcript abundance in brain zone 2 (telencephalon and olfactory bulbs removed; A,B) and the whole brain (C) were determined using a one-way ANOVA followed by a Tukey's *post hoc* test (*P*<0.05). Within a panel, groups that do not share a common letter are significantly different from one another.

*mr* mRNA abundance in brain zone 2 was significantly different among time points following acute stress exposure (*F*_4,28_=3.513, *P=*0.0191; Fig. 6A). After 6 h recovery, *mr* mRNA levels were 1.7-fold lower than pre-stress levels (*P*=0.0137) but recovered to baseline by 24 h. In contrast, *mr* mRNA abundance within brain zone 1 was not significantly affected by exposure to an acute stressor (*H*=2.081, *P=*0.5558, d.f.=3; Fig. S5C). Repeat acute stress significantly affected *mr* mRNA abundance in brain zone 2 (*F*_2,18_=8.521, *P*=0.0025; Fig. 6B), where a significant 1.6-fold reduction in *mr* RNA levels occurred at 15 min and 24 h recovery, relative to baseline (*P*=0.0067). In contrast, *mr* mRNA abundance in brain zone 1 was not significantly affected by repeat acute stress exposure (*F*_2,6=_2.611, *P*=0.1530; Fig. S5D), nor did social status affect whole-brain *mr* mRNA levels (*F*_2,19_=0.7530, *P*=0.4850; Fig. 6C).

## DISCUSSION

This study demonstrates the dynamic and stressor-specific regulation of Hsd11b2 expression in the zebrafish brain. An acute increase in cortisol coincided with a sustained upregulation of brain Hsd11b2 expression at the transcript and protein level for more than 24 h after the stress. Under the same time frame, a repeated acute stressor blunted this response while a chronic increase in cortisol failed to elicit any changes in brain Hsd11b2 expression. Importantly, the changes in Hsd11b2 elicited by acute stress coincided with reduced *gr* and *mr* expression as well as increased brain cell proliferation. These results suggest that intracellular cortisol signalling capacity can be tempered through a combination of increased cortisol inactivation and receptor downregulation. In turn, these changes would temporarily spare the inhibitory effects of stress on neurogenesis and may even potentiate pro-neurogenic signalling. Thus, this study supports Hsd11b2 as an agent for mediating stress-specific changes in brain cell proliferation.

### Stressor-specific regulation of *hsd11b2* expression in the adult zebrafish brain

Acute and chronic stressors induce characteristic but distinct changes in circulating glucocorticoids (Madliger and Love, 2014; Romero and Gormally, 2019). In zebrafish, a substantial increase in cortisol is observed within minutes of a fish experiencing an acute stressor, which then returns to basal levels within hours of recovery (Ramsay et al., 2009; Fuzzen et al., 2010; Alderman and Vijayan, 2012). This is consistent with the results of the present study, where plasma cortisol was significantly elevated 15 min after a brief air exposure and fully recovered by 24 h. This cortisol profile was exactly replicated when the stressor was repeated 1 day later, hence the designation of these regimes as acute and repeat acute stressors, respectively. In contrast, chronic stressors that persist for hours or days are frequently associated with sustained increases in circulating cortisol that last at least until the stressor abates. Dyadic fish pairings can serve as a chronic stressor in laboratory studies owing to the rapid establishment of distinct, robust behavioural phenotypes and elevated cortisol in socially subordinate fish throughout the interaction (Øverli et al., 1999; Sloman et al., 2001; Alderman et al., 2008; Bernier et al., 2008; Filby et al., 2010; Tea et al., 2019; but see Höglund et al., 2001; Pavlidis et al., 2011; Sørensen et al., 2012; Jeffrey and Gilmour, 2016). In the present study, cortisol levels were higher in subordinate relative to dominant male zebrafish after 24 h of continuous interaction; hence, this regime was designated a chronic stressor.

The distinct cortisol profiles under acute and chronic stress scenarios are thought to underlie the biphasic outcomes of elevated cortisol (Herman, 2013). For example, whereas acute stressors stimulate energy mobilization (Mommsen et al., 1999; Schreck and Tort, 2016), chronic stressors can result in prolonged catabolic responses that lead to growth inhibition (Sadoul and Vijayan, 2016). While this framework applies to many known actions of cortisol, including neurogenesis (Sørensen et al., 2013; Saaltink and Vreugdenhil, 2014), the cellular mechanisms that underpin such biphasic effects are not fully understood. Cortisol catabolism by Hsd11b2, which is irreversible in fish (Tsachaki et al., 2017), may play a role. At the organismal level, the absence of Hsd11b2 activity induced by pharmacological inhibition (Alderman and Vijayan, 2012) or CRISPR/Cas9-mediated knockout (Theodoridi et al., 2021) increased basal cortisol levels and delayed cortisol recovery following acute stress, highlighting a key role for Hsd11b2 in the physiological response to stress.

Stress-induced changes in *hsd11b2* expression in zebrafish have been observed in several cortisol target tissues including the head kidney (Fuzzen et al., 2010), gill and ovary (Lim and Bernier, 2022), as well as the brain (Alderman and Vijayan, 2012; Madaro et al., 2016; Huang et al., 2019). Nevertheless, there is considerable variation in the nature of this response, no doubt influenced by within-study restrictions on tissue/temporal sampling and across-study differences in stressor application. For example, Fuzzen et al. (2010) tracked *hsd11b2* mRNA levels in the head kidney of zebrafish during a 1 h agitation stress and 20 min recovery and showed that the transient increase in gene expression closely aligned with the cortisol profile in these fish; however, these temporal changes were reported within the context of a single stressor and tissue. Lim and Bernier (2022) demonstrated stressor- and tissue-specific changes in *hsd11b2* mRNA abundance in the ovaries and gills of zebrafish; however, measurements were taken at a single time point following chronic exposure to cycling hypoxia, temperature or combined stressors. The present study advances our understanding of the dynamic changes in Hsd11b2 expression associated with stressor-specific cortisol profiles and includes responses at both mRNA and protein levels. Specifically, we show that recovery from an acute stressor was marked by a persistent upregulation of Hsd11b2 gene and protein expression in the brain for at least 48 h, supporting the previously reported elevation in Hsd11b2 enzymatic activity in zebrafish brains 24 h after experiencing a single air exposure (Alderman and Vijayan, 2012). Intriguingly, this upregulation in Hsd11b2 expression was quickly suppressed in fish that experienced a second air exposure stressor, as both *hsd11b2* mRNA and Hsd11b2 protein abundance returned to baseline levels after 24 h recovery from repeat acute stress, the temporal equivalent of 48 h recovery in the single acute stress experiment. Indeed, the apparent increase in Hsd11b2 protein abundance at 15 min post-repeat acute stress may simply be a relic of the initial air exposure (temporal equivalent of 24 h recovery from acute stress). In contrast, the chronic stress of social subordination imposed a modest increase in brain Hsd11b2 expression at the gene but not protein level, aligning with previous work in rainbow trout after 96 h of social subordination (Best et al., 2023). Taken together, these results support rapid, experience-based changes in the capacity of the brain to catabolize intracellular cortisol and, subsequently, regulate downstream effects of cortisol signalling.

### A mechanism for regulating the effects of stress on adult neurogenesis

Hsd11b2 is well placed to contribute to context-specific regulation of adult neurogenesis by cortisol in zebrafish, given its localization to neurogenic niches (Alderman and Vijayan, 2012) and the stressor-induced changes in its expression (present study; Alderman and Vijayan, 2012; Best et al., 2023). Here, we provide novel support for this role by combining evidence of temporal changes in cortisol and Hsd11b2 expression with measures of brain cell proliferation. Consistent with the purported pro-neurogenic effects of acute stress in mammals (Thomas et al., 2006; Dagyte et al., 2009; Kirby et al., 2013; So et al., 2017) and teleost fish (von Krogh et al., 2010; Johansen et al., 2011; Zhang et al., 2020), we show that the expression of *pcna* in zone 1 and zone 2 of the brain increases within 15 min of recovery from acute stress, concomitant with elevated cortisol. There were also more BrdU+ cells in the telencephalon 1.5 h after air exposure, confirming a link between *pcna* expression and mitosis; confocal imaging and niche-specific cell counts are needed to resolve whether the increase in cell proliferation is a generalized or localized response. Notably, the increase in brain Hsd11b2 protein expression (present study) and activity (Alderman and Vijayan, 2012) that was also induced by acute stress was delayed relative to the cortisol response, suggesting that the early aftermath of stress is marked by a limited capacity to buffer intracellular cortisol levels in the brain. Given that repeat exposure to the acute stressor did not further promote brain cell proliferation, we propose that the increased capacity for cortisol catabolism realized by elevated Hsd11b2 prevented a second pro-neurogenic response in the repeat acute stress group.

Under chronic stress, brain *pcna* expression was unchanged in fish following 24 h of social subordination. This result was unexpected, given that decreased *pcna* expression was observed in the hypothalamus, optic tectum and telencephalon of rainbow trout following 72 h of social stress (Johansen et al., 2012). Similarly, fewer BrdU+ cells were observed in the telencephalons of subordinate male zebrafish that were injected with the mitotic marker after 24 h of subordination and euthanized 1 day later (Tea et al., 2019). In mammals, *pcna* expression has been shown to be highly transient during the cell cycle, with increased expression during S-phase followed by an immediate decline in M-phase (Ino and Chiba, 2000). In our acute stress experiment, we also found that *pcna* expression declined within hours of the initial increase. Thus, single time point assessment of subordinate fish may be insufficient to capture a change in *pcna* expression. Nevertheless, the absence of an Hsd11b2 response under chronic stress, as reported here, is consistent with our hypothesis. With a limited or status quo capacity to catabolize cortisol, intracellular cortisol in NSPCs could reach or exceed the threshold required to induce the anti-neurogenic response that is often observed in socially subordinate teleosts (Johansen et al., 2012; Tea et al., 2019).

Variation in receptor abundance is another mechanism by which context-specific responses to cortisol can be realized in target tissues. At the same time, Gr and Mr have different ligand binding affinities in fish (Bury et al., 2003; Greenwood et al., 2003; Sturm et al., 2005), as is seen in mammals (Koning et al., 2019). Thus, while both Gr and Mr are widely expressed in the fish brain (Wendelaar Bonga, 2011), the relative proportion of high-affinity Mr to low-affinity Gr may also influence the nature of cortisol signalling in target cells. Indeed, stress is known to impart brain region-specific changes in *gr* and *mr* mRNA abundance (Johansen et al., 2011; Alderman and Vijayan, 2012; Pavlidis et al., 2015; Madaro et al., 2016; Molteson et al., 2016; Kiilerich et al., 2018; Best et al., 2023). In rainbow trout selected for distinct stress coping styles, fish characterized by a strong cortisol response to acute stress had lower *mr* but similar *gr1* and *gr2* expression across several brain regions compared with fish characterized by a low cortisol response to acute stress (Johansen et al., 2011). These differences in receptor expression are thought to explain, at least in part, the distinct behavioural and physiological phenotypes of these fish (Johansen et al., 2011), including stress-induced changes to *pcna* expression in the telencephalon (Johansen et al., 2012). In the present study, there was a consistent downregulation of brain *gr* and *mr* expression after both acute and repeat acute stress exposure, but no changes under chronic stress. Thus, although the two cortisol receptors did not show differential responses, these results support receptor abundance as a mechanism for stressor-specific effects of cortisol in the brain. Interestingly, the timing of receptor expression changes was delayed relative to the initial cortisol response following air exposure, as occurred with Hsd11b2; however, *gr* and *mr* downregulation persisted during repeat acute stress whereas Hsd11b2 recovered. Taken together, these results support experience-based changes in the capacity of NSPCs for cortisol catabolism and receptor-mediated signalling.

The present study used homogenates that contained multiple brain regions, and thus provides a holistic assessment of how variation in cortisol catabolism, alongside receptor abundance, can contribute to stressor-specific responses in the brain. Mitotic cell labelling and BrdU+ cell counts in the telencephalon begin to connect these changes to neurogenesis; however, additional studies are needed to understand niche-specific responses to stress in the forebrain, and whether these changes in brain cell proliferation alter neuronal populations. Thus, an important next step is to resolve the cellular phenotypes that express Hsd11b2 in the zebrafish brain. In the context of adult neurogenesis, for example, knowing which NSPC populations and/or neuronal lineages express Hsd11b2 will help disentangle the mechanisms that underpin stressor-specific changes to neuronal cell populations. Indeed, there is growing evidence that the many neurogenic niches in the zebrafish brain are diversely and uniquely regulated by endogenous factors and environmental stimuli (Caron et al., 2022). Ultimately, this will aid in understanding the biological significance of adult neurogenesis.

### Conclusions

Stress imparts myriad effects on animal physiology, including changes in the brain. Here, the influence of cortisol on the cell cycle can alter neural circuits that are populated by new neurons derived from adult NSPCs, providing a mechanism for experience-based learning. In animals with a high regenerative capacity, such as the zebrafish, regulating the influence of cortisol on this process may be necessary to strike a balance between continuity and plasticity in the neural circuitry of the brain. This study presents evidence that Hsd11b2 is differentially regulated in the zebrafish brain under acute and chronic stress. We propose the Hsd11b2 response as a novel mechanism through which NSPCs may modulate rates of cortisol catabolism and, subsequently, context-specific changes to cell proliferation in the post-natal brain. While we provide clear evidence that Hsd11b2 is regulated by stress, it remains unknown whether the changes reported in this study are themselves mediated by increased cortisol or by alternative pathways. In support of the former, putative glucocorticoid response elements have been identified in the promotor regions of both mammalian and zebrafish *hsd11b2* genes (Surjit et al., 2011; Alderman and Vijayan, 2012). However, other molecular and cellular signalling pathways such as DNA methylation, microRNAs and the p38 mitogen-activated protein kinase (P38 MAPK) pathway may also play a role in regulating zebrafish *hsd11b2*, as they do in mammals (Sharma et al., 2009; Peña et al., 2012; Rezaei et al., 2014). Studies that increase our understanding of the stress-specific pathways controlling the regulation of Hsd11b2 will provide further insight into how zebrafish utilize endogenous regulators to directly mediate changes to adult neurogenesis and, subsequently, help elucidate how neuroplasticity is maintained throughout their life.

## Supplementary Material

10.1242/jexbio.248020_sup1 Supplementary information
