# Supplementary material for: 11β-Hydroxysteroid dehydrogenase type 2 may mediate the stress-specific effects of cortisol on brain cell proliferation in adult zebrafish (Danio rerio)
Source: J Exp Biol. 2024 Aug 27;227(16):jeb248020. doi: 10.1242/jeb.248020 (PMC11418181; doi:10.1242/jeb.248020)
Supplement: Supplementary information [file jexbio-227-248020-s1.pdf]

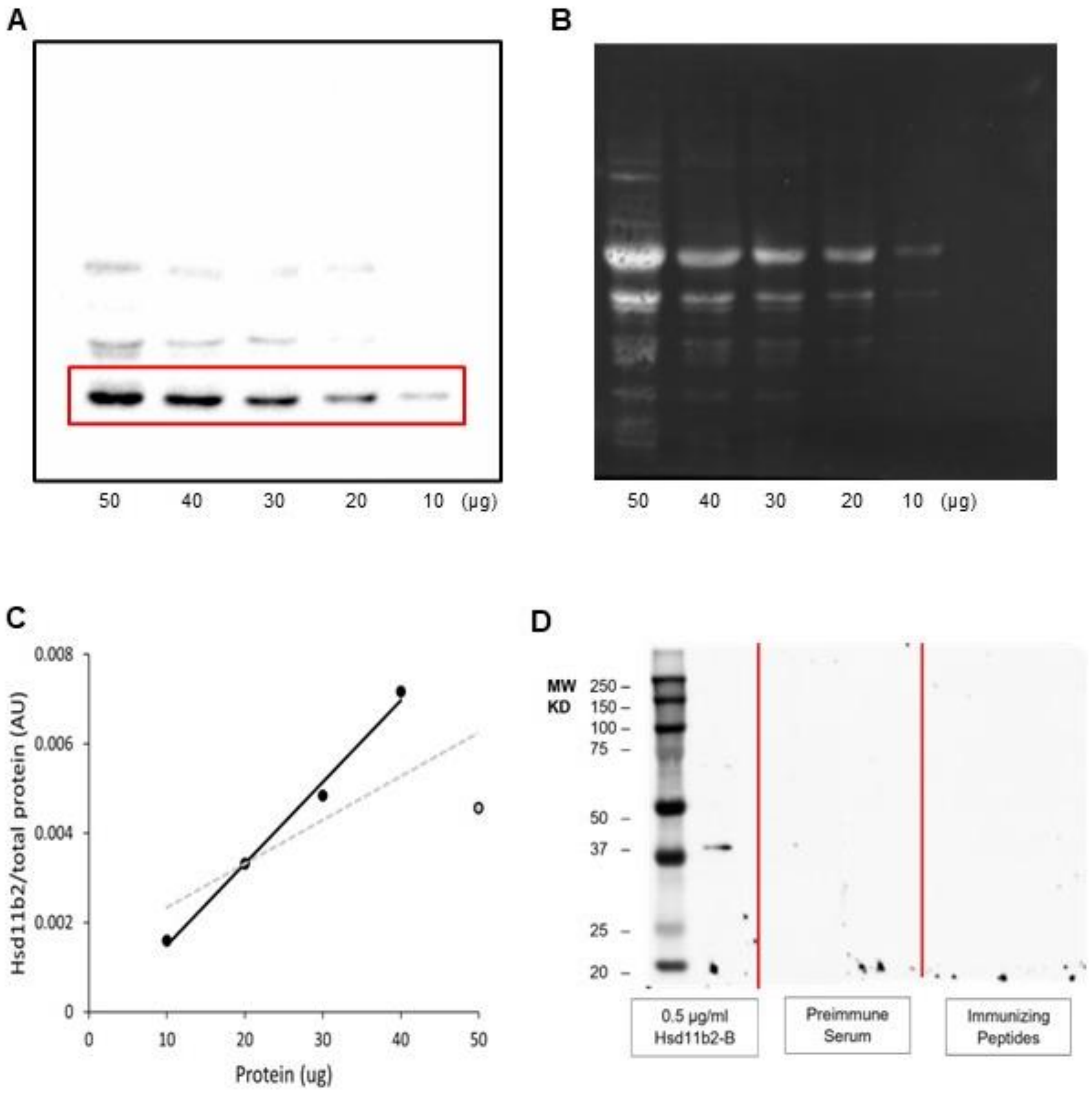

Journal of Experimental Biology • Supplementary information

Fig. S1. (A) A western blot where increasing amounts of zebrafish brain homogenate (10 - 50 µg) were loaded on 10% SDS-PAGE gel and transferred to a nitrocellulose membrane. ECL detection of Hsd11b2 (bands outlined by red rectangle) using the chicken-anti-Hsd11b2 antibody. **(B)** Fluorescent detection of total protein on the same blot using SYPRO Ruby Red Protein Blot Stain. **(C)** A linear regression analysis of Hsd11b2/total protein band intensity in proportion to increased protein loading showing the linear range of detection, represented by the black trendline (10 - 40 µg, R^2 = 0.9913), and that above 40 µg, the signal begins to plateau and sample loading does not exhibit the expected increase in band intensity, represented by the grey dashed trendline (10 - 50 µg, R^2 = 0.5673). **(D)** A western blot where 20 µg aliquots of zebrafish brain homogenate were loaded into each lane and then divided into 3 pieces following transfer (red line). From left to right, the split blots were incubated in 0.5 µg ml^{-1} chicken-anti-Hsd11b2 (38-KDa), pre-immune serum, and 0.5 µg mL^{-1} chicken-anti-Hsd11b2 containing 5 µg mL^{-1} immunizing peptide. There was no immunoreactivity observed in the pre-immune or immunizing peptide sections proving the specificity of the chicken-anti-Hsd11b2 antibody.

Journal of Experimental Biology • Supplementary information

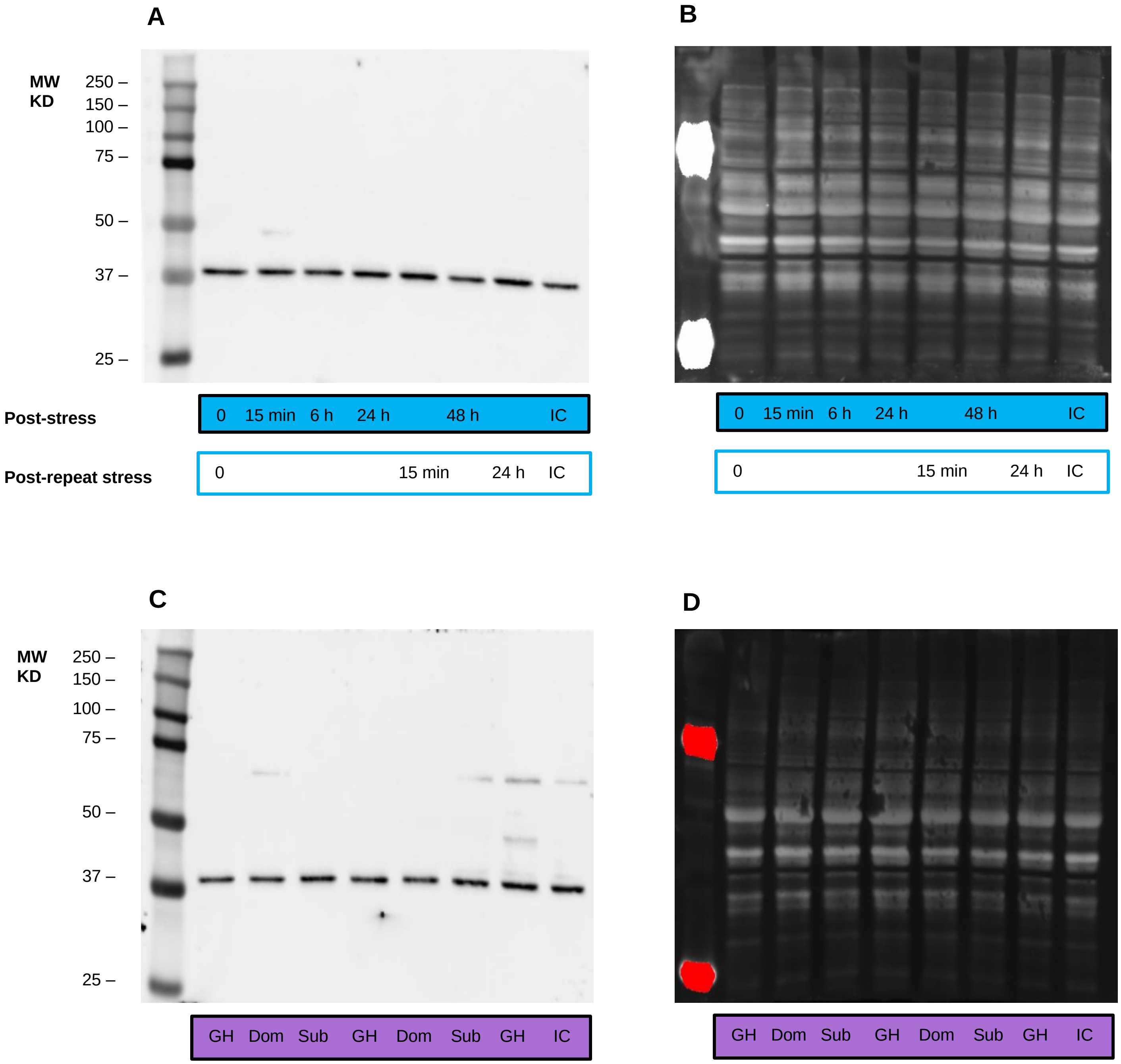

Fig. S2. Representative western blots showing **(A)** Hsd11b2 protein expression determined using chicken-anti-Hsd11b2 antibody, and **(B)** total protein determined using SYPRO Ruby Red Protein Blot Stain following exposure to a single (solid blue) and repeat (blue outline) 1-min air exposure stressor and recovery in the brains of adult zebrafish. Representative western blots of **(C)** Hsd11b2 protein expression determined using chicken-anti-Hsd11b2 antibody, and **(D)** total protein determined using SYPRO Ruby Red Protein Blot Stain following 24 h of social stress in the brains of dominant (Dom) and subordinate (Sub) zebrafish; group-housed (GH) fish are included as a control. IC = internal control from pooled brain homogenates.

Journal of Experimental Biology • Supplementary information

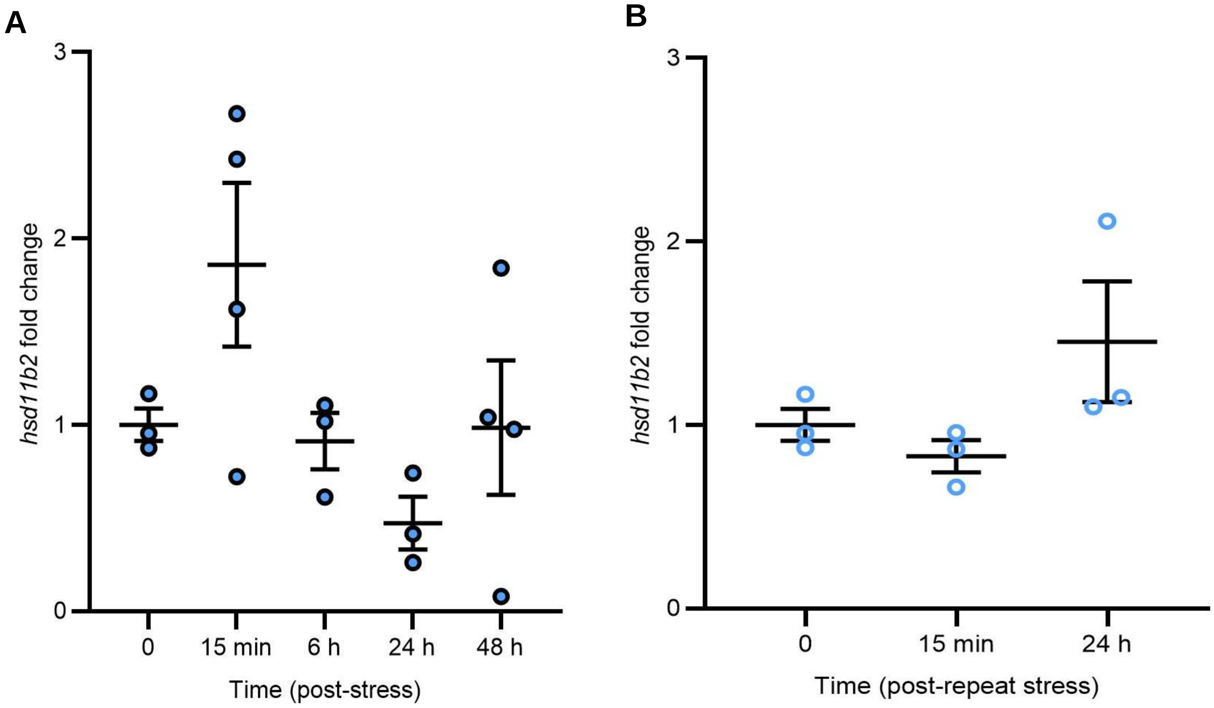

Fig. S3. Effects of an acute (closed blue circles) and repeat (open blue circles) 1-min air exposure stressor on *hsd11b2* transcript abundance in brain zone 1 (telencephalon, olfactory bulbs; n=2 fish pooled per sample). **(A)** Pooled brain zone 1 *hsd11b2* transcript abundance after exposure to an acute, and **(B)** repeat acute stressor. Gene transcript abundance is normalized to the mean expression of two housekeeping genes (*ef1α* and *rpl8*). Data is presented as individual data points, with solid lines and whiskers representing the mean ± SEM for each timepoint (*hsd11b2* transcript abundance acute stressor n = 3-4, repeat acute stressor n = 3). Differences in *hsd11b2* mRNA levels following acute and repeat acute stress exposure were determined using a one-way ANOVA with a Tukey's *post-hoc* test ($P > 0.05$).

Journal of Experimental Biology • Supplementary information

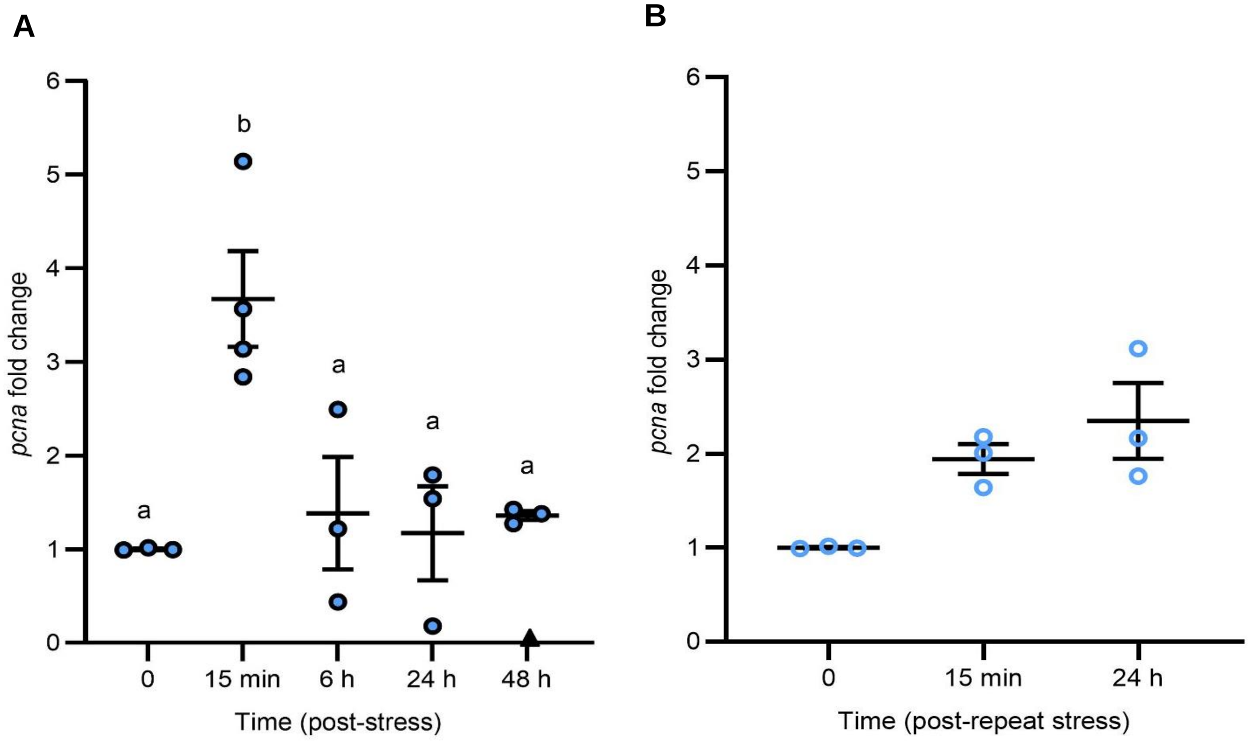

Fig. S4. Effects of an acute (closed blue circles) and repeat (open blue circles) 1-min air exposure stressor on *pcna* transcript abundance in the pooled brain zone 1 (telencephalon, olfactory bulbs; n=2 fish pooled per sample). **(A)** Pooled brain zone 1 *pcna* transcript abundance after exposure to an acute, and **(B)** repeat acute stressor. Gene transcript abundance is normalized to the mean expression of two housekeeping genes (*ef1α* and *rpl8*). Data is presented as individual data points, with solid lines and whiskers representing the mean ± SEM for each timepoint (*pcna* transcript abundance acute stressor n = 3-4, repeat acute stressor n = 3). Outliers removed from treatment groups represented by black triangles. Differences in *pcna* mRNA levels following acute ($P < 0.05$) and repeat acute stress ($P > 0.05$) exposure were determined using a Kruskal Wallis test followed by Dunn's *post-hoc* test. Letters indicate significant differences among groups within a panel; groups which have the same letter are not significantly different from one another.

Journal of Experimental Biology • Supplementary information

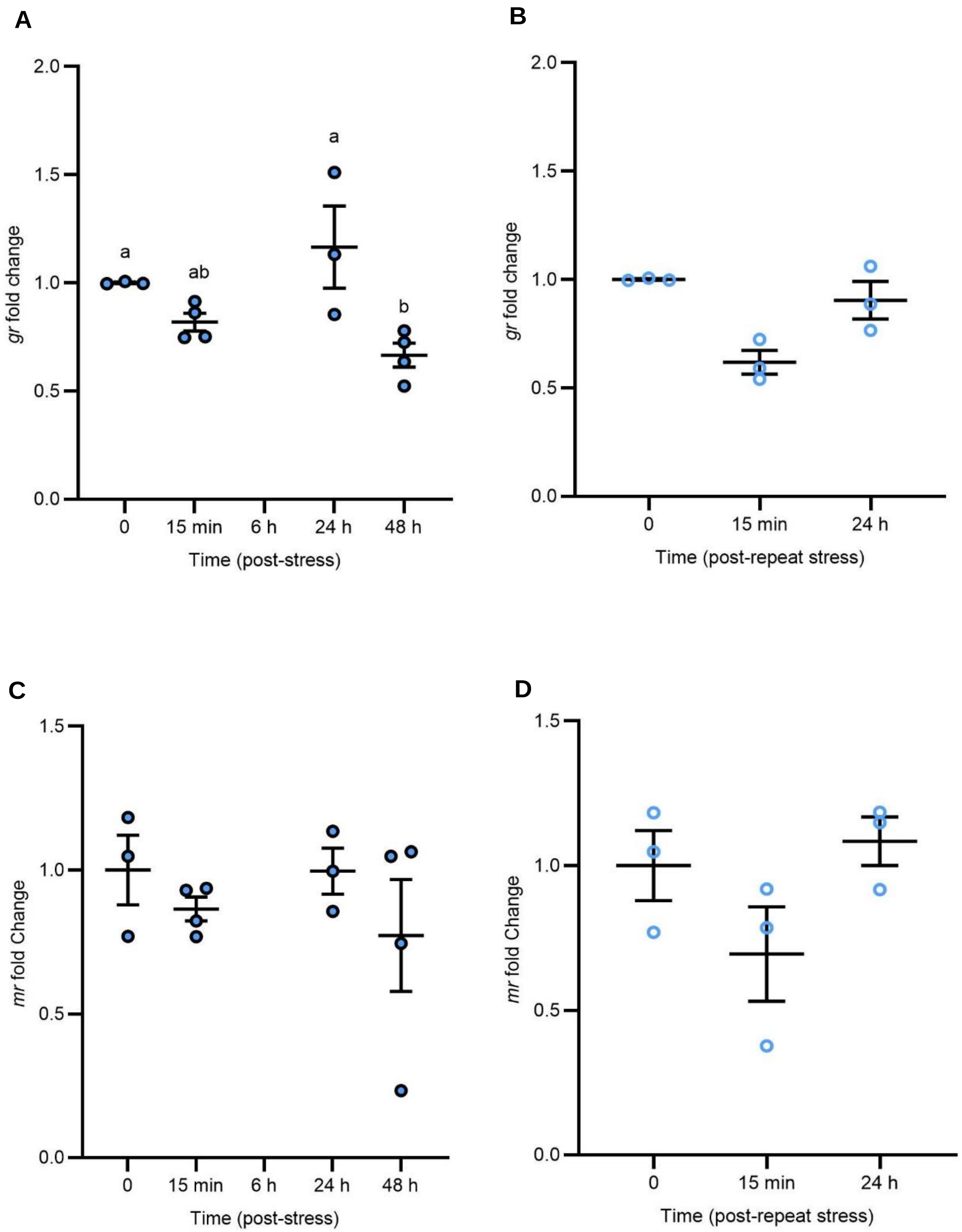

Journal of Experimental Biology • Supplementary information

Fig. S5. Effects of an acute (closed blue circles) and repeat (open blue circles) 1-min air exposure stressor on *gr* and *mr* transcript abundance in the pooled brain zone 1 (telencephalon, olfactory bulbs; n=2 fish pooled per sample). **(A)** Pooled brain zone 1 *gr* transcript abundance after exposure to an acute, and **(B)** repeat acute stressor. **(C)** Pooled brain zone 1 *mr* transcript abundance after exposure to an acute, and **(D)** repeat acute stressor. Gene transcript abundance is normalized to the mean expression of two housekeeping genes (*ef1α* and *rpl8*). Data is presented as individual data points, with solid lines and whiskers representing the mean ± SEM for each timepoint (*gr* transcript abundance acute stressor n = 2-4, repeat acute stressor n = 3; *mr* transcript abundance acute stressor n = 2-4, repeat acute stressor n = 3). The 6 h post-stress recovery timepoint is omitted for *gr* and *mr* mRNA abundance due to an insufficient sample size. Differences in *gr* and *mr* mRNA levels following acute and repeat acute stress exposure were determined using a Kruskal Wallis test followed by Dunn's *post-hoc* test except for acute stress *mr* expression where a one-way ANOVA followed by Tukey's *post-hoc* test was used. Letters indicate significant differences among groups within a panel; groups which have the same letter are not significantly different from one another.

Journal of Experimental Biology • Supplementary information
